# Enrichment of In Utero Rhythmic Experience Promotes Postnatal Visuospatial Attention

**DOI:** 10.1111/nyas.70441

**Published:** 2026-10-26

**Authors:** Martina Arioli, Valentina Silvestri, Melissa Savoldi, Niccolò Giovannini, Maria Lorella Giannì, Lorenzo Colombo, Viola Macchi Cassia

**Affiliations:** ^1^ Department of Psychology University of Milano‐Bicocca Milan Italy; ^2^ Department of Obstetrics and Gynecology Fondazione IRCCS Ca’ Granda Ospedale Maggiore Policlinico Milan Italy; ^3^ Department of Clinical Sciences and Community Health University of Milan Milan Italy; ^4^ NICU Fondazione IRCCS Ca' Granda Ospedale Maggiore Policlinico Milan Italy

**Keywords:** infants, in utero experience, neuroplasticity, newborns, prenatal stimulation, rhythm, visuospatial attention

## Abstract

By 2 months of age, infants leverage predictable temporal structures to optimize attention allocation, disengaging faster from rhythmic than nonrhythmic stimuli—a pattern absent in newborns and attributed to postnatal experience. Here, we investigated whether prenatal rhythmic enrichment provides an earlier foundation for this competence. From week 29 until delivery, pregnant women (*N* = 101) provided fetuses either multisensory rhythmic stimulation (experimental, *N* = 49) or nonrhythmic relaxation (control, *N* = 52). Visual attention disengagement was assessed using an overlap task with rhythmic, nonrhythmic, and static stimuli, at birth (*N* = 30 per group) and 2 months (*N* = 30 per group). At 2 months, both groups showed the expected attentional cost for nonrhythmic stimuli. At birth, this pattern appeared only in the experimental group, indicating that prenatal rhythmic enrichment accelerates the typical developmental timeline for temporal attention tuning. Individual differences further showed that greater amounts of prenatal rhythmic stimulation predicted faster overall disengagement at birth, and a stronger cost for nonrhythmic stimuli at 2 months. While acknowledging methodological constraints inherent to the population under study, these findings suggest that the developing brain begins to tune to environmental temporal structure prenatally, and that this process is promoted by in utero rhythmic experience.

## Graphical abstract

From week 29 until delivery, pregnant women exposed their fetuses to either multisensory rhythmic stimulation or nonrhythmic relaxation. Visual attention disengagement was assessed at birth and 2 months using rhythmic, nonrhythmic, and static stimuli. At birth, only rhythm‐enriched newborns showed the attentional cost for nonrhythmic stimuli observed at 2 months in both groups. Greater prenatal rhythmic exposure was associated with faster disengagement at birth and a stronger cost for nonrhythmic stimuli at 2 months. These findings suggest that prenatal rhythmic experience promotes early tuning of attention to environmental temporal structure.

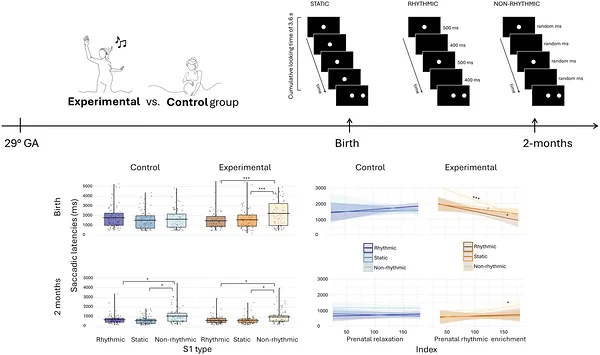

## 1 Introduction

Human cognition exploits the regular temporal structure of dynamic environmental events to optimize information processing. This regularity, frequently operationalized as rhythm—patterns of regular events in time separated by equal (isochronous) or unequal (anisochronous) intervals [1]—supports predictive processing, facilitating attention allocation, and enhancing information‐processing speed and accuracy [2].

The link between environmental rhythms and attention is formally captured by the Dynamic Attending Theory (DAT; [3]). DAT posits that rhythm allows the cognitive system to build temporal expectations and dynamically allocate resources to high‐probability points in time. This rhythmic guidance optimizes outcomes across auditory and visual domains (e.g., Refs. [4, 5]). For example, adults exhibit significantly shorter saccadic latencies when targets appear in synchrony with a predictable rhythmic beat compared to an unpredictable one [6].

Rhythm is ubiquitous across the human lifespan. From birth, infants encounter dense, multimodal rhythmic input through dyadic interactions—like infant‐directed speech, singing, and rocking (e.g., Refs. [7, 8])—to which the postnatal brain and behavior readily entrain (e.g., Refs. [9, 10, 11]). However, intense exposure to rhythmic cues begins in utero, long before birth [12]. By the third trimester, the fetus is sensitive to the rhythmic patterning of vestibular [13] and auditory [14] information transmitted through the maternal body. The auditory system, in particular, becomes functional from approximately 24–28 weeks of gestation [15], enabling the processing of abstract temporal structures [16, 17]. Accordingly, fetuses can discriminate between rhythmically distinct languages [18] and musical tempos [14].

Given this dense in utero exposure and the high cortical plasticity [19] characterizing this developmental window, one could hypothesize that the cognitive system learns to harness rhythm already prenatally, with the effects of this adaptation manifest at birth. This perspective conceptualizes the link between rhythm and attention as an emerging property of neurodevelopment [20], an adaptive response to the temporal structure of sensory input, guaranteed by the interplay between brain plasticity and environmental structure during the perinatal period. Supporting this, 3‐month‐old infants produce significantly more anticipatory eye movements toward visual events whose timing is predictable [21], and 2‐month‐olds synchronize their gaze behavior to the rhythm of infant‐directed singing while looking at the caregiver's face [10]. Notably, Arioli et al. [22] showed that 2‐month‐olds disengage visuospatial attention faster from rhythmic versus nonrhythmic stimuli, an effect that is absent in newborns. This developmental shift was attributed to the accumulation of postnatal multimodal experience with rhythm especially available during multimodal face‐to‐face interactions. Accordingly, postnatal rhythmic training has proven effective in promoting temporal processing of music and speech [23], sociocommunicative skills in typically developing infants [24], language acquisition in children with developmental language disorder [25], and interpersonal synchrony in children with autism spectrum disorder [26].

In the current study, we extended this approach to the prenatal period to investigate whether prenatal rhythmic multisensory enrichment could provide an earlier foundation for the development of postnatal visuospatial attention, by experimentally manipulating the fetal sensory environment in utero. Specifically, we examined whether such prenatal stimulation is effective in promoting the ability to leverage visual temporal patterns for attentional allocation, a competence that typically emerges at around 2 months of age but is absent at birth [22].

To address this question, we recruited pregnant women to provide multisensory rhythmic enrichment (experimental group) or nonrhythmic relaxation (control group) to their fetuses from the 29th week of gestation until delivery (see Ref. [27] for a description of the intervention protocol). Then, using the same visual overlap task utilized by Ref. [22], we measured infants’ visual attention disengagement at birth and 2 months of age. Gaze‐shift accuracy and latency toward a peripheral target (S2) were recorded as a function of the central stimulus’ (S1) temporal characteristics (rhythmic, nonrhythmic, stationary). We hypothesized that, at birth, only prenatally enriched newborns would show slower disengagement from nonrhythmic stimuli—a pattern typically absent until 2 months [22]. At 2 months, we expected both groups to exhibit this pattern, reflecting typical development, though potentially more pronounced in the experimental group. Finally, we also conducted exploratory analyses to examine whether variability in the amount of prenatal enrichment and postnatal exposure to rhythmic patterns modulated individual infants’ postnatal attentional performance.

## 2 Materials and Methods

### 2.1 Study Design

The present study employed a prospective, randomized intervention design to investigate how enriched prenatal multisensory rhythmic stimulation influences infants’ attentional disengagement at birth and 2 months postpartum. Starting from the 29th week of gestation, pregnant women delivered either a rhythmic or a nonrhythmic intervention to their fetuses. Participants were randomly assigned to one of two groups: the experimental or control group. At the time of recruitment, mothers were informed of their group assignment, received instructions for the corresponding protocol, and were told that infants' visual behavior would be assessed at birth and again at 2 months. The study's specific hypotheses were disclosed at each testing session (at birth and at 2 months), as part of the informed consent procedure for the behavioral assessment. Mothers in the experimental group were instructed to actively engage in multisensory rhythmic activities by listening to a predefined playlist of pop music while simultaneously singing or humming, and moving and tapping their belly (regardless of fetal position) along with the rhythm for at least 15 min, four times per week. This multisensory approach was designed to facilitate the extraction of rhythmic information through intersensory redundancy [28]. Mothers in the control group followed an identical schedule but were instructed to passively listen to soothing nature sounds while seated or lying down. The song length did not differ between the experimental (M = 248.2 s, SD = 30.2) and the control (M = 261.25 s, SD = 182) groups, *t*(20.05) = 0.32, *p* = 0.75.

To monitor protocol adherence, mothers completed weekly diaries to rate the frequency with which they performed each prescribed activity for their respective groups (humming, moving, and tapping for the experimental group; level of relaxation for the control group) using 5‐point Likert scales. Composite scores derived from these measures indicated robust compliance, as evidenced by the high mean scores across both groups (experimental group: M = 12.95/15, SD = 1.9; control group: M = 3.31/5, SD = 0.45. See Table S1 for details). Mothers also recorded the number of songs or tracks played per session to provide a quantitative proxy of total stimulation for use in statistical analyses. On average, mothers from the experimental group played 11.57 songs per week (SD = 2.99), while mothers from the control group played 14.4 relaxing sounds per week (SD = 6.36). We additionally assessed whether mothers engaged in other rhythm‐related activities during the third trimester independent of the experimental protocol (e.g., yoga, gym, aquagym, dancing, swimming); the prevalence of such activities did not differ between groups (see Table 1). A comprehensive description of the protocols pipeline, including the selection of auditory stimuli and diary items, is provided in Ref. [27].

**TABLE 1:** Summary of participants’ demographics.

	Experimental group	Control group	Statistical significance
*Mothers*	Mean (SD)	Mean (SD)	*p* value
Age	36 (4.76)	34.8 (4.98)	0.27
Education^a^	5.43 (1.13)	5.53 (1.17)	0.73
Occupation^b^	4.26 (0.76)	3.82 (1.03)	0.06
Activities involving music during last trimester	*n*	*n*	
Yoga/meditation	8	7	
Gym	5	4	
Aquagym	3	1	0.83
Swimming	7	9	
Dancing	5	6	
*Newborns*	Mean (SD)	Mean (SD)	*p* value
Gestational age (weeks)	39.47 (1.28)	39.86 (1.34)	0.22
Age (h)^c^	84.4 (75)	75.1 (52.3)	0.59
Birth weight (g)	3094.52 (363.03)	3235.36 (360.45)	0.53
	*n*	*n*	
Female	13	10	0.5
Apgar at 1 and 5 min	9, 10	9, 10	—
*2‐month‐old infants*	Mean (SD)	Mean (SD)	*p* value
Age (days)^c^	77.4 (32.8)	75.4 (16.4)	0.78
	*n*	*n*	
Female	12	14	0.22

^a^ Maternal education was scored on a 7‐point scale: (1) primary school; (2) junior high school; (3) vocational degree; (4) high school; (5) bachelor's degree; (6) 5‐year master's degree; (7) doctoral degree.

^b^ Maternal employment was scored on a 5‐point scale: (1) occupations with minimal skills, no qualifications, or advanced training; (2) occupations that require basic training or role‐specific skills; (3) occupations requiring an intermediate technical qualification; (4) occupations requiring a moderate level of qualification and specialized training; (5) highly qualified occupations.

^c^ See Supporting Information file for control analyses including exact age as covariate.

### 2.2 Participants

Participants were recruited on a voluntary basis through childbirth education classes at the Obstetrics and Gynecology Unit of Fondazione IRCCS Ca' Granda Ospedale Maggiore Policlinico of Milan, located in an urban environment. Following an initial email invitation sent by the hospital, interested women attended an online meeting during which they were provided with an overview of the study's aims and procedures. They then completed an online screening survey (Qualtrics) to provide contact information and medical history. Inclusion criteria required participants to be Italian‐speaking, over 18 years of age, and experiencing an uncomplicated pregnancy. Eligible women were randomly assigned to either the experimental or control group. Upon assignment, participants provided informed consent and received the materials required for their respective protocols. The final sample included 101 mother–infant dyads (experimental group, *N* = 49; control group, *N* = 52). They all came from middle‐class families and their parents self‐reported as White. Due to high participant drop‐out and attrition rate, the study adopted a mixed design: 60 infants were tested at birth (30 per group) and 60 were tested at 2 months (30 per group); of these, 19 were tested at both time points (11 experimental, 8 control), while the remaining participants provided data for a single time point. Participants’ demographics are summarized in Table 1. An additional 67 infants (42 at birth, 25 at 2 months) were recruited but excluded from the final analysis; detailed exclusion criteria are provided in Table S2. The target sample size was informed by Ref. [22], which conducted 1000 Monte Carlo simulations based on a pilot sample (*N* = 6) with the same study design. That analysis indicated that a sample of 30 participants per group would be sufficient to provide a power of 82.4%. To further validate this, we performed a sensitivity analysis using the SIMR package for R [29] to determine the smallest detectable effect with 80% power and alpha = 0.05 using mixed effect models. This was not an observed retrospective power analysis; rather, we systematically varied the effect sizes of the *S1_type × Group × Age* fixed effects of interest for our hypothesis [30]. Results confirmed that our sample of 101 participants (contributing 984 observations) was sufficient to detect a difference of 810 ms for the effect of interest.

Neonatal testing sessions were carried out at the Neonatal Intensive Care Unit of Fondazione IRCCS Ca' Granda Ospedale Maggiore Policlinico of Milan, while the 2‐month sessions took place at the Bicocca Child and BabyLab of the University of Milano‐Bicocca. Across sites, testing conditions were comparable. All infants were tested when awake and in an alert state. The protocol met the ethical standards of the Declaration of Helsinki and was approved by the relevant Ethics Committee (Comitato Etico Milano Area 2; ID: 694; Approval No. 952_2021). Written informed consent for infants’ participation in the study was obtained at each testing session (at birth and at 2 months) from all parents.

### 2.3 Stimuli

Stimuli for both S1 (central cue) and S2 (peripheral target) consisted of white geometric shapes presented against a black background, generated using E‐Prime 2.0. While S2 always remained static, the temporal dynamics of S1 were manipulated to create three experimental conditions: a static condition where S1 remained stationary on the screen, a rhythmic condition where S1 flickered according to a 500 ms on–400 ms off temporal pattern, resulting in a frequency of ∼1.11 Hz, and a nonrhythmic condition where S1 flickered without any discernible pattern (i.e., on–off phases ranging between 200–3000 ms and between 200–800 ms). The average presentation rate was approximately 1.15 Hz, effectively matching the overall stimulation rate of the rhythmic condition while eliminating temporal predictability.

For the newborns sample, the stimuli consisted of three distinct geometric shapes (full circle, empty circle, and 2 × 2 checkerboard), each subtending 13° × 13° of visual angle when viewed from a distance of 30 cm (S1–S2 inner distance was 22.62°). For the 2‐month‐old sample, a fourth geometric shape was introduced to maintain stimulus counterbalancing despite the lower trial count (see Procedure section). Given the increased viewing distance for older infants (50 cm), stimuli subtended 8° × 8° of visual angle, with an S1–S2 inner distance of 13.68°.

### 2.4 Apparatus and Procedure

Infants were tested in a dimly lit, quiet room. Newborns were seated on the experimenter's lap at a viewing distance of approximately 30 cm from the monitor, while 2‐month‐old infants were seated on a caregiver's lap at a distance of 50 cm. An experimenter monitored the infant's visual behavior via a real‐time video feed from a camera positioned above the screen to initiate each trial. The video was recorded using a mini‐Dv digital recorder to allow for the subsequent offline coding of saccadic latencies to reach S2.

Testing began when the baby was alert and fixated on the screen. Each trial started with the onset of the central stimulus (S1) in one of three conditions: static, rhythmic or nonrhythmic. Once the experimenter online coded 3.6 s of continuous fixation on S1, the peripheral target (S2) appeared randomly on either the left or right side of S1, while S1 remained visible on the screen. In the rhythmic and nonrhythmic S1 conditions, the onset of S2 was strictly synchronized with the appearance of S1 (Figure 1). Both stimuli remained on the screen until the infant performed a saccadic eye movement to disengage from S1 or until 10 s had elapsed. At the end of each trial, a central fixation cross was presented to redirect the infant's attention back to the center of the screen. Once the infant fixated the center, a new S1 was displayed and the next trial began, for a total of 12 trials.

**FIGURE 1:**
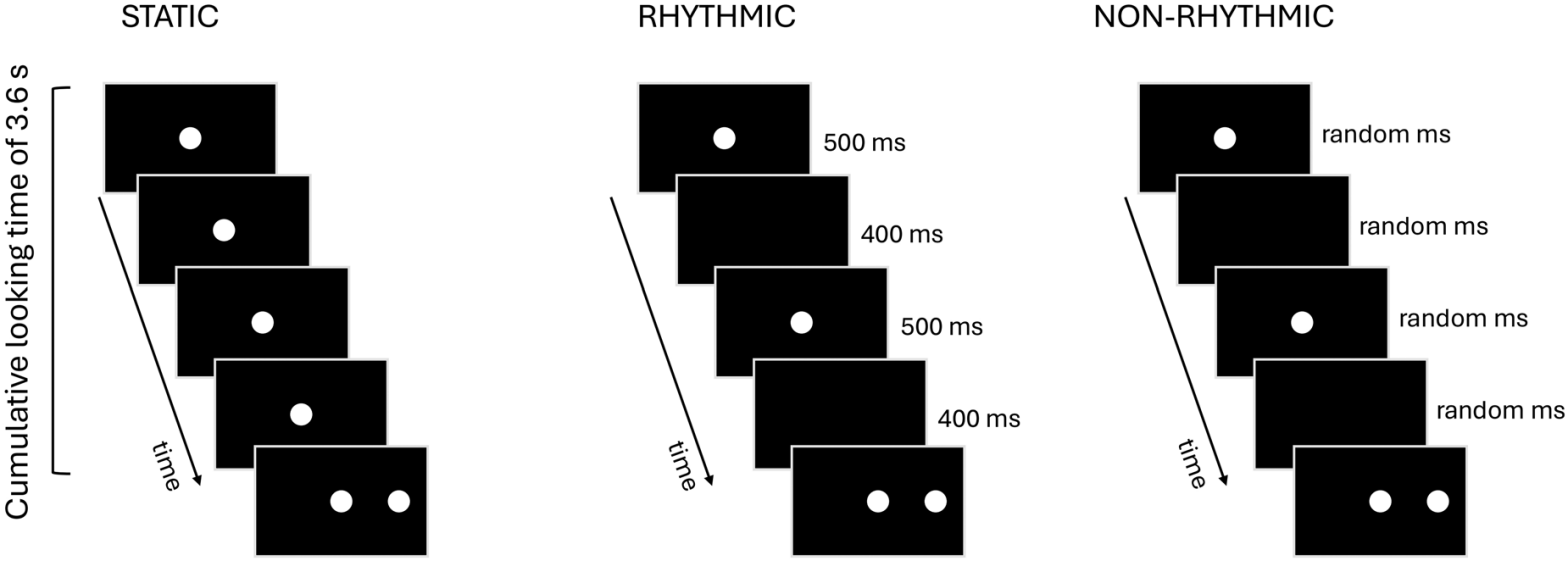
Trial structure for each S1 Type condition of the overlap task (after Ref. [22]) presented to newborns and 2‐month‐old infants.

These 12 trials were grouped into three 4‐trial blocks, with the S1 Type condition (rhythmic, static, nonrhythmic) held constant within each block. Blocks sequence was counterbalanced across participants using two block orders: rhythmic–static–nonrhythmic (experimental group: *N* = 29; control group: *N* = 33), or nonrhythmic–static–rhythmic (experimental group: *N* = 31; control group: *N* = 27). Given the high variability of neonatal neurobehavioral states (e.g., Ref. [31]), newborns were presented with three additional two‐trial blocks, one for each S1 Type condition, to increase the likelihood of obtaining a number of trials comparable to that of the 2‐month‐old infants. The order of these additional blocks followed the initial assignment, beginning with the S1 Type condition presented in the final block of the first set. Trial presentation continued until the infant became fussy or inattentive or until they completed the task.

### 2.5 Prenatal Intervention Metrics and Postnatal Environmental Experience Measures

To account for interindividual variability in prenatal intervention metrics and postnatal rhythmic experience, we derived a series of composite indices, used as potential moderators in exploratory analyses. Descriptive statistics for these measures are provided in Table S1.

#### 2.5.1 Prenatal Intervention Metrics

Data regarding the quantity of the prenatal stimulation provided to the fetuses were extracted from the weekly diaries completed by the mothers:

• Prenatal rhythmic enrichment index: As a proxy for the cumulative amount of prenatal rhythmic stimulation experienced by the fetus, we calculated the total number of songs played by the mothers of the experimental group throughout the protocol period.

• Prenatal relaxation index: To provide a parallel control metric for the rhythmic enrichment index, we calculated the total number of soothing nature sounds utilized by mothers in the control group over the same protocol duration.

#### 2.5.2 Postnatal Exposure Metrics

Although the intervention concluded at birth, we collected data on the infants’ naturalistic exposure to rhythmic patterns during the first 2 months of postnatal life to control for environmental influences. Mothers completed a questionnaire at the 2‐month testing session, reporting the frequency of music listening (on a 5‐point Likert scale: 1 = *never*, 5 = *almost every day or always*) and the typical duration of these sessions (in minutes). When music was played, mothers also rated how often they engaged in singing or dancing with their infants. Based on these metrics, we derived the following postnatal index:

• Postnatal rhythmic exposure index: A composite index calculated as the mean of the products of the normalized frequency ratings and the normalized duration of listening, singing, and dancing sessions. This multiplicative approach was used to ensure that the index reflects both the regularity and the intensity of the musical environment, preventing a high frequency of very short sessions from being overweighted against longer, more sustained interactions. All variables were normalized (scaled from 0 to 1) prior to composite calculation to ensure equal weighting.

### 2.6 Data Reduction

Infant gaze behavior was coded offline by two independent coders who were blind to the study's hypotheses. Coding was performed using VirtualDub, a video capture and processing utility for 64‐bit Windows platforms, at a frame rate of 25 frames per s (40 ms resolution). For each trial, coders measured the cumulative fixation time on S1 to ensure inclusion criterion was met, as well as the saccadic latency to S2. Saccadic latency was defined as the interval between the onset of S2 and the initiation of a gaze shift toward the target. Trials with coding discrepancies exceeding 40 ms were jointly recoded. If a consensus could not be reached, the trial was excluded (this occurred in only 1.07% of trials, *N* = 5 in the newborn sample).

To account for age differences in attention and motor control, we applied age‐specific criteria for trial validity. A trial was considered valid if the infant: (1) accumulated at least 2.7 s of continuous looking toward S1 (corresponding to three full repetitions of the rhythmic pattern); (2) maintained stable fixation on S1 for at least 500 ms right before S2 onset; (3) initiated a saccade toward S2 within a valid temporal window (200–10,000 ms for newborns; 200–5000 ms for 2‐month‐olds); and (4) directed the saccade straight to S2 without intervening look‐aways or blinks. At the participant level, newborns were included in the final analysis if they contributed at least two valid trials in at least two of the three S1 Type conditions. Due to the increased attentional stability expected at 2 months, 2‐month‐old infants were required to contribute at least two valid trials in all three S1 Type conditions to be included.

### 2.7 Statistical Analyses

Statistical analyses were performed in RStudio (RStudio Team, 2022). We used linear mixed‐effects models (LMMs) to account for both interindividual variability and the hierarchical structure of the data, with repeated observations nested within participants.

Separate models were conducted for the number of valid trials and the saccadic latencies. For both dependent variables, the model included S1 Type (rhythmic, nonrhythmic, static), group (experimental, control), age (newborns, 2 months), and their interactions as fixed predictors, and a by‐subject random intercept to account for individual differences in the performance. In addition, separate models were run for each age group, including exact age as a covariate (in hours for newborns and in days for 2‐month‐olds; see Supporting Information file for details). To assess whether the observed effects on saccadic latency could be driven by differences in the number of valid trials, we conducted an additional LMM using mean saccadic latency as the dependent variable. S1 Type, group, age, and their interactions were included as fixed effects, with the number of valid trials entered as a covariate and a by‐subject random intercept. Results are shown in the Supporting Information file.

To investigate how individual differences in the amount of prenatal rhythmic enrichment influenced newborns’ and 2‐month‐olds’ saccadic latencies, we ran separate models for the experimental and the control groups. These models included the indices derived from the weekly prenatal diaries (prenatal rhythmic enrichment index, prenatal relaxation index) as covariates, together with S1 Type, Age, and their interactions as fixed predictors and a by‐subject intercept for participants.

Furthermore, to assess the impact of postnatal rhythmic exposure on the saccadic latencies of 2‐month‐old infants across the three S1 Type conditions, we conducted a model with only 2‐month‐old infants’ data, with S1 Type, Group, postnatal rhythmic exposure index, and their interactions as fixed predictors and a by‐subject intercept for participants.

The normality of the dependent variables was assessed using Shapiro–Wilk tests. Saccadic latencies exhibited a non‐normal distribution (all *W*s < 0.89, *p*s < 0.001). Additionally, the residuals of the models were not normally distributed and exhibited heteroscedasticity, as shown by the Q–Q plot of residuals and the Residuals versus Fitted plot. We also assessed the presence of influential outliers by computing Cook's distance and found that all values remained well below commonly used thresholds of concern (e.g., Di < 1 or the more conservative 4/*n*), with a maximum value of approximately 0.06. LMMs are reported to be robust to violations of normality, particularly with the inclusion of random effects (e.g., Ref. [32]), and have been successfully applied to neonatal datasets (e.g., Ref. [33]). However, to ensure robustness of our conclusions, we replicated all latency analyses using log‐transformed saccadic latencies [34], with results reported in the Supporting Information. The log transformation substantially improved distributional properties, yielding approximately normally distributed and homoscedastic residuals. Cook's distance values remained low (all Di < 0.04), further confirming the absence of influential outliers.

In addition to parameter estimation, Bayesian hypothesis testing was performed using Bayes factors (BFs). BFs were computed using the Savage–Dickey density ratio by comparing posterior distributions to corresponding prior distributions. Weakly informative Cauchy priors (location = 0, scale = 0.707) were specified for fixed effects. For each contrast of interest, BFs were estimated by comparing posterior estimates with prior distributions obtained from prior predictive sampling. Posterior sampling was performed using four Markov chain Monte Carlo chains with 40,000 iterations each. Prior predictive sampling was enabled to allow Bayesian hypothesis evaluation.

All data and R‐codes used for the analyses are publicly available at https://osf.io/nr45a/overview?view_only=c7af696d71984ef78ab3f58a9892bbb4.

## 3 Results

Average valid trials and saccadic latencies toward S2 for each S1 Type condition, group, and age group are detailed in Table 2.

**TABLE 2:** Mean (SD) number and percentage of valid trials, and mean (SD) saccadic latency (ms), as a function of S1 Type, group, and age group.

Measure	S1 Type	Experimental group	Control group
**Birth**			
Number of valid trials	Static	2.62 (0.73)	3.39 (1.23)
	Rhythmic	2.71 (0.81)	2.79 (0.92)
	Nonrhythmic	3 (0.92)	2.86 (0.93)
% valid trials	Static	43.66%	56.6%
	Rhythmic	45.17%	46.5%
	Nonrhythmic	50%	47.67%
Saccadic latency (ms)	Static	1575 (1011)	1532 (955)
	Rhythmic	1477 (909)	1789 (1088)
	Nonrhythmic	2229 (1310)	1636 (979)
**2 months**			
Number of valid trials	Static	3.07 (0.74)	2.93 (0.83)
	Rhythmic	2.77 (0.73)	3 (0.79)
	Nonrhythmic	2.93 (0.74)	2.77 (0.82)
% valid trials	Static	76.75%	73.25%
	Rhythmic	69.25%	75%
	Nonrhythmic	73.25%	69.25%
Saccadic latency (ms)	Static	638 (362)	657 (393)
	Rhythmic	660 (425)	740 (451)
	Nonrhythmic	1024 (666)	1113 (787)

### 3.1 Number of Valid Trials

The analysis of the number of valid trials per participant revealed a significant three‐way interaction between S1 Type, group, and age, *F*(2,234.39) = 3.54, *p* = 0.031. Bonferroni‐corrected simple effects indicated that for the newborn control group only, the number of valid trials was significantly higher in the static S1 Type condition (M = 3.39, SE = 0.18) than in both the rhythmic (M = 2.79, SE = 0.16, *t*(248) = 2.74, *p* = 0.02), and nonrhythmic conditions (M = 2.85, SE = 0.16, *t*(246) = 2.47, *p* = 0.043; Figure 2). No other group or age comparisons reached significance (all *p*s > 0.26).

**FIGURE 2:**
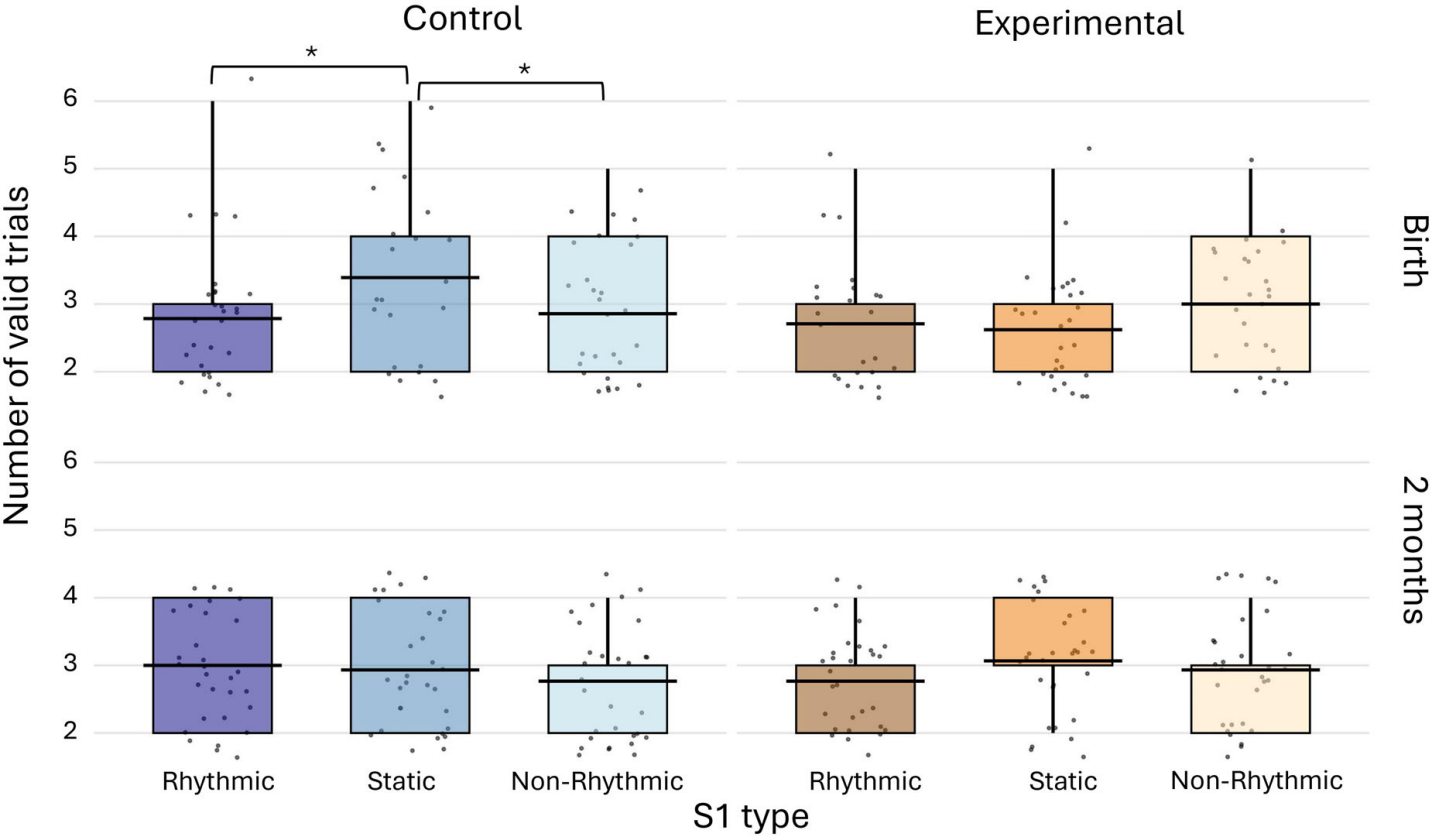
Box plots represent the number of valid trials for newborns and 2‐month‐old infants across the three S1 types, categorized by group. Black horizontal lines indicate the means, while the colored boxes indicate the interquartile range (IQR). Dots display estimated individual data points. *p < 0.05.

Bayesian analyses did not provide converging evidence in favor of the triple interaction (BFs = 0.15–0.46), instead indicating that the data were more likely under the null hypothesis. Full BF results for all model parameters are reported in the Supporting Information file.

### 3.2 Saccadic Latencies

The LMM for saccadic latencies revealed significant main effects of S1 Type, *F*(2,903.78) = 23.31, *p* < 0.001, and age, *F*(1,298.05) = 226.12, *p* < 0.001. These were qualified by a significant S1 Type × Group interaction, *F*(2,903.78) = 6.09, *p* = 0.002, which was further superseded by a significant three‐way interaction between S1 Type, group, and age, *F*(2,903.62) = 7.1, *p* < 0.001. Bonferroni‐adjusted simple effects showed that, at 2 months, both groups were slower to disengage visual attention from the nonrhythmic S1 stimuli (M = 1048 ms, SE = 67.6) compared to the static (M = 634 ms, SE = 66.2) and rhythmic (M = 686 ms, SE = 67.4) stimuli (all *p*s < 0.01). However, at birth, this pattern was exclusive to the experimental group. Newborns in this group exhibited significantly slower attentional disengagement in the nonrhythmic S1 Type condition (M = 2218 ms, SE = 98.4) compared to both the static (M = 1581 ms, SE = 100.1, *t*(905) = 5.06, *p* < 0.001) and rhythmic (M = 1480 ms, SE = 107.7, t(922) = 5.6, *p* < 0.001) conditions (Figure 3). No significant differences between conditions were observed for newborns in the control group (all *p*s > 0.17). Between‐group comparisons revealed that the experimental and control groups did not differ significantly in any condition at 2 months (all *p*s = 1.00). In contrast, at birth a significant group difference emerged exclusively in the nonrhythmic S1 condition, where newborns in the experimental group showed significantly slower latencies compared to controls (*p* < 0.001). These results were consistent when analyzed using log‐transformed latencies (see Supporting Information file) with the exception of the between‐group difference in the nonrhythmic S1 condition at birth, which was no longer significant after log transformation (all *p*s > 0.18). Importantly, the supplementary analysis that included the number of valid trials as a covariate suggests that these results were not driven by differences in the number of valid trials contributing to each condition: the critical S1 Type × Group × Age interaction remained significant, and the number of valid trials did not significantly predict saccadic latency (*p* = 0.63; see Supporting Information file).

**FIGURE 3:**
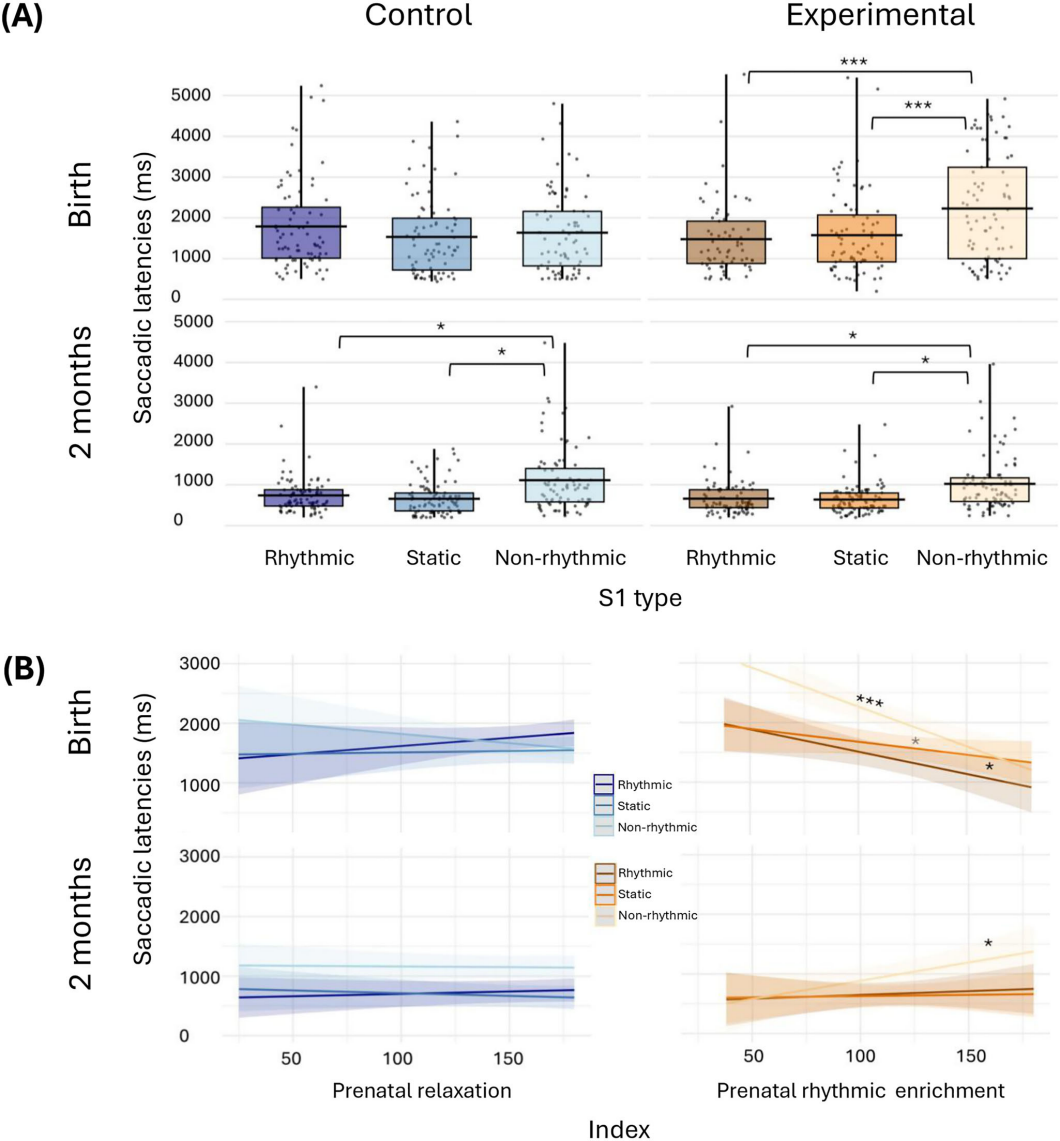
Panel A depicts the box plots of saccadic latencies for newborns (top) and 2‐month‐old (bottom) infants across the three S1 Type conditions, categorized by group. Black lines indicate the means, the colored boxes represent the interquartile range (IQR). Estimated individual data points are displayed as dots. Panel B illustrates the saccadic latencies across the three different S1 Type conditions for newborns (top) and 2‐month‐old (bottom) infants in the control and in the experimental groups plotted as a function of the prenatal relaxation index and prenatal rhythmic enrichment index. *p < 0.05; ***p ≤ 0.001.

Bayesian analyses provided converging evidence with the frequentist results. In line with the LMM findings, strong BFs supported the main effects of S1 Type (BFs = 114–> 1000) and Age (BF > 1000), as well as their interaction (BFs = 45.96–29.59). Evidence for the three‐way interaction (S1 Type × Age × Group) was also observed, although weaker (BFs ≈ 0.64–1.33). Nonetheless, planned comparisons further confirmed the frequentist patterns. At 2 months, there was strong evidence for differences between the nonrhythmic and both the rhythmic (control: BF = 76.59; experimental: BF ≈ 1.12 × 10^3^) and static (control: BF ≈ 1.04 × 10^4^; experimental: BF ≈ 7.27 × 10^3^) conditions in both groups. In newborns, strong evidence for differences between nonrhythmic and both rhythmic (BF = 18.58) and static conditions (BF = 17.55) was observed exclusively in the experimental group. In contrast, for the control group comparisons between S1 Type conditions yielded BFs below 1 (BFs = 0.07–0.14), providing evidence in favor of the null hypothesis of no reliable differences between stimulus types. Similarly, no evidence for differences between rhythmic and static conditions was found in the experimental group (BF = 0.014). Regarding between‐group comparisons, consistent with the log‐transformed frequentist analysis, no substantial evidence in favor of group differences was found across conditions at either timepoint (all BFs < 0.907). Complete BF results for all model parameters are reported in the Supporting Information file.

### 3.3 Exploratory Effects of Prenatal Intervention Metrics

For the experimental group, the prenatal rhythmic enrichment index significantly influenced saccadic latencies, *F*(1,43.36) = 4.5, *p* = 0.039. This effect interacted with age, *F*(1,162.83) = 22.43, *p* < 0.001, and further participated in a three‐way interaction with S1 Type, *F*(2,383.38) = 4.66, *p* = 0.01. Bonferroni‐corrected simple effects showed that, for newborns, higher prenatal cumulative exposure to rhythmic enrichment was associated with faster disengagement (negative slopes) in the rhythmic (*b* = −7.47, *t*(261) = −2.74, *p* = 0.007) and nonrhythmic (*b* = −13.28, *t*(231) = −5.06, *p* < 0.001) S1 Type conditions, with a marginal trend for the static condition (*b* = −4.35, *t*(230) = −1.76, *p* = 0.07). At 2 months, this trend reversed specifically for the nonrhythmic condition (*b* = 6.14, *t*(248) = 2.07, *p* = 0.039; Figure 3). No significant effects of the prenatal relaxation index were found in the control group (all *p*s > 0.1). The analyses performed on log‐transformed data confirmed this pattern of results (see Supporting Information file).

For the experimental group, Bayesian analyses provided substantial evidence against a main effect of the prenatal rhythmic enrichment index on saccadic latencies (BF = 0.066). Similarly, there was no evidence for interactions between the prenatal index and S1 Type (BFs = 0.007–0.028), nor for the three‐way interaction involving prenatal index, S1 Type, and age (BFs = 0.010–0.052). In contrast, there was substantial evidence for an interaction between the prenatal rhythmic enrichment index and age (BF = 25.69), driven by a negative association at birth (median = −0.0049, 95% HPD [−0.0075, −0.0023]) and no evidence of an effect at 2 months (median = 0.0026, 95% HPD [−0.0004, 0.0054]). For the control group, Bayesian analyses were consistent with the absence of effects of the prenatal relaxation index on saccadic latencies (all BFs < 1/3). The full BFs are reported in the Supporting Information file.

### 3.4 Exploratory Effects of Postnatal Exposure Metrics

Exploratory models assessing the potential impact of the postnatal rhythmic exposure index on 2‐month‐old infants’ attentional disengagement performance yielded no significant effects (all *p*s > 0.27). These null findings were consistent in the models on the log‐transformed saccadic latencies (all *p*s > 0.27; see Supporting Information file).

BFs provided no evidence in favor of any effect, with weak to inconclusive evidence for an interaction between the postnatal rhythmic exposure index and S1 Type (BFs = 0.448–1.68). Simple slope analyses confirmed BFs below 1, supporting the absence of any reliable effect. The full BFs are reported in the Supporting Information file.

## 4 Discussion

In line with our hypothesis, prenatal enrichment of the in utero rhythmic experience promoted infants’ postnatal ability to exploit temporal patterns during the allocation of visuospatial attention. Unlike newborns in the control group, who showed no significant difference in disengagement between rhythmic and nonrhythmic conditions—consistent with Arioli et al. [22]—enriched newborns exhibited slower saccadic latencies to disengage attention to nonrhythmic stimuli. Because the control group's performance mirrored the pattern observed in newborns without prenatal intervention [22], the performance shift in the experimental group is specifically attributable to the enrichment protocol.

The trial validity data further reflected the potential impact of prenatal enrichment. Frequentist analyses showed that for the newborns in the control group only, the number of valid trials was significantly higher in the static condition compared to both the rhythmic and nonrhythmic conditions. This pattern in the control group is consistent with the notion that static stimuli are less demanding to process, whereas the flickering inherent to dynamic displays might introduce greater oculomotor interference (e.g., premature saccades) or fatigue in nonstimulated newborns, leading to more excluded trials. The absence of this pattern in the experimental group suggests that prenatal rhythmic stimulation may have enhanced infants' processing efficiency of dynamic visual inputs, smoothing the differences in trial validity across conditions. However, this interpretation must be treated with caution, as Bayesian analyses did not provide converging evidence for these comparisons, which indicates that this specific effect on trial numbers is relatively weak.

This increased efficiency likely explains why the performance of enriched newborns mirrored that observed in 2‐month‐olds across both groups. This finding suggests that the link between rhythm and attention is not driven solely by biological maturation—for example, neural entrainment or tracking of external periodic stimuli [35]—but is instead a plastic adaptation of the developing brain to the dynamic temporal structure of sensory input. Crucially, our results suggest that this adaptation begins prenatally, during the third trimester of gestation, and is significantly promoted by enriching the fetus’ rhythmic environment through active maternal engagement.

Our finding that, in the enriched newborns and 2‐month‐olds alike, nonrhythmic stimuli imposed a cost on attention disengagement parallels adult data showing that saccadic latencies are significantly reduced when a target appears in synchrony with a rhythmic auditory beat compared to a random beat [6]. Current results extend this evidence to newborns and the visual modality, showing that at the start of postnatal life, the absence of discernible regularity in dynamic visual input imposes a measurable cost on attention allocation. Future studies manipulating the temporal synchrony between central and peripheral stimuli are needed to determine whether this cost stems from the failure to leverage attentional alignment with the S1's temporal structure, or from a higher information‐processing load imposed by the inherent unpredictability of the nonrhythmic information flow.

Consistent with [22], saccadic latencies did not differ between rhythmic and static S1 conditions, both of which elicited faster disengagement than the nonrhythmic S1. This likely reflects the fact that both possess low entropy and high predictability—through periodic recurrence and temporal persistence, respectively—allowing the system to minimize uncertainty, and more efficiently allocate attention resources to the incoming information.

A finding that may appear inconsistent with the present results is that prenatal stimulation might have been expected to reduce saccadic latencies to S2 in the rhythmic S1 condition in the experimental group relative to the control group. Although our results do not support this conclusion, this plausibly reflects a combination of methodological constraints. First, our analyses may have had limited sensitivity to detect small‐to‐moderate effects, given the relatively low number of valid trials, even if this number remains comparable to those reported in previous studies [22, 36, 37]. Moreover, average saccadic latencies in our control group were already comparatively fast (1664 ms) relative to those reported in previous newborn studies using comparable disengagement paradigms, including Refs. [22] (1918 ms, using the same stimuli as in the current study), [36] (2024 ms, using flashing‐light cues/targets), and [37] (2500–3200 ms, using schematic faces). This suggests that our paradigm may already elicit comparatively rapid disengagement at baseline, possibly approaching a physiological floor for saccadic latency that would limit the detectability of further reductions following prenatal enrichment. Future studies should attempt to replicate these findings using larger samples and tasks that are more sensitive to detect such differences.

The finding emerging from group analyses that prenatal stimulation affected postnatal disengagement performance was complemented by the individual difference analyses. According to frequentist analysis, and with partial support from Bayesian analyses, the cumulative amount of prenatal rhythmic enrichment predicted faster overall disengagement in newborns, regardless of S1 Type, suggesting a generalized enhancement of attentional processing speed at birth. This aligns with evidence from avian embryos where prenatal rhythmic stimulation enhances postnatal visuospatial attention [38], an effect linked to mitigated stress responses, as evidenced by decreased posthatch corticosterone levels, and augmented neural plasticity [39]. Given that this effect was specific to rhythmic prenatal stimulation and not to relaxing stimulation, it is possible that rhythmic stimulation prenatally trains temporal prediction mechanisms that are also recruited during attentional disengagement, such that fetuses exposed to a predictable rhythmic structure become more efficient at shifting attention away from a no‐longer‐informative stimulus. At 2 months, the impact of prenatal stimulation on individual‐level performance appeared to be more specific, as higher prenatal stimulation predicted a significantly greater cost in disengagement efficiency from nonrhythmic stimuli, suggesting stronger reliance on rhythmic predictability. This shift may suggest that early prenatal experience exerts a lasting influence on how infants prioritize temporal cues that likely interact with and is refined by subsequent postnatal rhythmic exposure. However, this finding warrants cautious interpretation, as Bayesian analyses did not provide converging evidence supporting a reliable effect at this stage.

Interestingly, postnatal rhythmic exposure did not show similar associations, with BFs indicating evidence in favor of the absence of an effect. One possibility is that, by 2 months, postnatal rhythmic input may be sufficiently rich to promote a stable behavioral pattern across all infants, leveling out individual variations. Alternatively, this null result may instead reflect measurement limitations. As postnatal exposure was not experimentally manipulated but assessed post hoc via proxy measures, our tools may lack the sensitivity to detect subtle interindividual differences. It is also worth noting that the postnatal enrichment scores were overall relatively low, which may have further limited our ability to detect associations with saccadic latency. In addition, the current measure of postnatal rhythmic exposure was restricted to the musical environment and therefore does not capture other ecologically valid sources of rhythmic stimulation, such as being rocked, bounced, or swayed, which may occur with or without accompanying speech, song, or music. Future studies employing more fine‐grained and comprehensive measures will be essential to disentangle how postnatal experience interacts with or adds to in utero exposure.

A critical aspect of the current findings is that all the observed postnatal effects of prenatal stimulation pertained to the visual modality, despite the enrichment intervention being delivered through auditory, vestibular, and tactile channels. This indicates that the fetal brain extracts predictable temporal structure as an amodal feature, utilizing cross‐modal bootstrapping processes where structured input in certain modalities supports the organization of attentional mechanisms in another. This is in line with behavioral evidence showing that tempo and rhythm are two amodal stimulus properties whose discrimination is initially facilitated by intersensory redundancy (e.g., when input variations occur simultaneously in multiple modalities) and eventually becomes consistent even in unimodal contexts (e.g., Ref. [28]). In our study, the multisensory nature of the prenatal intervention likely provided the redundancy necessary for the fetal brain to extract the rhythmic structure from the input, accelerating the developmental transition to the ability to extract and leverage rhythmic regularities from unimodal visual stimuli immediately upon birth.

### 4.1 Limitations

A major limitation of the present study is the relatively small number of valid saccadic latency trials, which likely reduces statistical power and thus the sensitivity to detect effects of small to moderate magnitude. Although both the number of valid trials and the observed saccadic latency values are comparable to those reported in the existing literature (e.g., Ref. [37]), suggesting that our data quality is consistent with standard practice in the field, the limited amount of usable data per condition may still have constrained our ability to identify subtle effects. Importantly, however, the number of valid trials did not significantly predict saccadic latency in our data, and the main pattern of results remained unchanged when it was included as a covariate (see Supporting Information file), suggesting that the reported effects are unlikely to be artifacts of trial availability.

Additionally, while we monitored and found no significant differences between groups regarding maternal exposure to structured nonprotocol‐related prenatal physical and rhythmic activities (e.g., dancing, yoga; see Table 1), a limitation of the present study is the lack of a comprehensive, standardized assessment of the broader prenatal auditory environment and parental musical background. Future research should expand this line of investigation by incorporating more detailed and comprehensive assessments, for example, through questionnaires and standardized scales, to capture ecological auditory experiences and formal parental musical expertise, as these factors may further modulate early neurocognitive sensitivity to rhythmic stimulation.

Finally, in the absence of an out‐of‐synchrony condition, the present findings remain inconclusive with respect to the specific mechanism underlying the observed effects—namely, whether they reflect disruption in the dynamic allocation of attention or a more general information‐processing cost imposed by the inherent unpredictability of the nonrhythmic information flow. Future studies shall address this issue by directly manipulating the synchronicity of the peripheral target appearance with respect to the rhythmic beat of the central cue.

## 5 Conclusion

To conclude, the present findings indicate that the ability to leverage temporal regularities to optimize attention allocation is an emerging property of human cognition, arising from the interaction between neurodevelopmental plasticity and early rhythmic experience. These results extend our understanding of the first 1000 days [40]—the period from conception to a child's second birthday—which represents a window of unparalleled opportunity and vulnerability in brain development. Because efficient attentional disengagement is fundamental to language acquisition and sociocognitive development [41], promoting this skill early in development is vital to fostering adaptive developmental trajectories. In light of the Antenatal Investment Hypothesis [42], which emphasizes the highest returns of early interventions beginning in utero, the finding that rhythmic enrichment during the third trimester promotes postnatal attention development, provides a robust scientific evidence base to inform programs aimed at fostering neurocognitive development and mitigating early risk factors.

## Author Contributions

**Martina Arioli**: conceptualization, methodology, software, validation, formal analysis, visualization, data curation, investigation, writing – original draft preparation, writing – reviews and editing; **Valentina Silvestri**: validation, investigation, data curation, writing – reviews and editing; **Melissa Savoldi**: investigation, data curation, writing – original draft preparation; **Niccolò Giovannini**: methodology, resources, writing – review and editing; **Maria Lorella Giannì**: methodology, resources, writing – review and editing; **Lorenzo Colombo**: methodology, resources, supervision, writing – review and editing; **Viola Macchi Cassia**: conceptualization, methodology, validation, resources, funding acquisition, supervision, project administration, writing – original draft, writing – reviewing and editing.

## Conflicts of Interest

The authors declare no conflicts of interest.

## Supporting information




**Supporting Information**: nyas70441‐sup‐0001‐SuppMat.docx

## Data Availability

The hypotheses, methods, and the analysis plan were not preregistered. The materials necessary to attempt to replicate the findings presented here are not publicly accessible, but available upon request to the first author. All primary data and analysis scripts are publicly available at the following link: https://osf.io/nr45a/overview?view_only=c7af696d71984ef78ab3f58a9892bbb4.
