# Supplementary material for: Enrichment of In Utero Rhythmic Experience Promotes Postnatal Visuospatial Attention
Source: Ann N Y Acad Sci. 2026 Oct 26;1564(1):e70441. doi: 10.1111/nyas.70441 (PMC13697228; doi:10.1111/nyas.70441)
Supplement: Supplementary file 1 [file NYAS-1564-0-s001.docx]

**Supplementary file**

**Enrichment of in-utero rhythmic experience promotes postnatal visuospatial attention**

Martina Arioli^1*^, Valentina Silvestri^1^, Melissa Savoldi^1^, Niccolò Giovannini^2^, Maria Lorella Giannì^3,4^, Lorenzo Colombo^4^**^+^**, Viola Macchi Cassia^1^**^+^**

^1^ Department of Psychology, University of Milano-Bicocca, Milan, Italy

^2^ Department of Obstetrics and Gynecology, Fondazione IRCCS Ca’ Granda Ospedale Maggiore

Policlinico, Milan, Italy

^3^ Department of Clinical Sciences and Community Health, University of Milan, Milan, Italy

^4^ NICU, Fondazione IRCCS Ca' Granda Ospedale Maggiore Policlinico, Milan, Italy

^*^ Corresponding author

**^+^** These authors have contributed equally to this work and share last authorship

The present file contains:

- Summary of participants’ prenatal intervention metrics and postnatal rhythmic exposure index (p. 2);
- Details on exclusion criteria (p. 3);
- Statistical analyses and results on the log-transformed saccadic latencies (p. 4);
- Analyses with exact age as covariate (p. 5);
- Analyses with number of valid trials as covariate (p. 6);
- Bayesian models and full Bayes factor tables (pp. 7-13).

**Participants’ prenatal intervention metrics and postnatal rhythmic exposure index**

**Table S1.** Summary of participants’ prenatal intervention metrics and postnatal rhythmic exposure index.

|  | Experimental Group | Control Group | Statistical significance |
| --- | --- | --- | --- |
|  | *Mean (SD; min-max)* | *Mean (SD; min-max)* | *p value* |
| Rhythmic protocol compliance* | 12.95 (1.9; 8.4-15) | *-* |  |
| Relaxing protocol compliance^+^ | *-* | 3.31 (.45; 2.33-4.02) |  |
| Prenatal rhythmic enrichment index^†^ | 112.56 (38.2; 38-191) | - |  |
| Prenatal relaxation index^†^ | - | 151.13 (68.4; 27-368) |  |
| Postnatal rhythmic exposure index^§^ | .1 (.8; .02-.35) | .17 (.2; .004-1) | .25 |

* Rhythmic protocol compliance: a composite score, ranging from 1 to 15, derived from averaging the weekly scores calculated by summing the ratings from the three 5-point Likert scales on which participants rated the frequency of the prescribed activities performed (singing/humming, moving, and tapping);

^+^ Relaxing protocol compliance: a score, ranging from 1 to 5, derived from averaging the weekly relaxation ratings across the protocol duration.

† Prenatal rhythmic enrichment and relaxation indices: a variable score indicating the total number of songs or soothing tracks played by mothers throughout the protocol period.

^§^Postnatal rhythmic exposure index: a score, ranging from 0 to 1, computed as the mean of the products of normalized frequency and duration ratings for music listening, singing, and dancing activities.

**Details about excluded participants**

**Table S2.1.** Details of excluded dyads - newborn sample.

|  | Experimental group | Control group |
| --- | --- | --- |
| Covid-19 | 2 | 1 |
| Fussiness | 8 | 8 |
| Insufficient number of valid trials | 8 | 6 |
| Prematurity | 1 | 2 |
| Intensive Care Unit | 3 | 3 |

**Table S2.2.** Details of excluded dyads - 2-month-old infant’s sample.

|  | Experimental group | Control group |
| --- | --- | --- |
| Fussiness | 5 | 6 |
| Insufficient number of valid trials | 6 | 5 |
| Technical errors | 2 | 1 |

**Statistical analyses on log-transformed saccadic latencies**

*lmer(log(SaccadicLatency)~ S1_Type*Group*Age+(1|ID), data=DB_all)*

Results showed significant main effects of S1_type, F(2, 287.7) = 31.75, p < .001 and Age, F(1, 393.23) = 270.52, p < .001, as well as significant S1_type*Group, F(2, 897.70) = 5.04, p = .006 and S1_type*Age, F(2, 897.27) = 6.01, p = .003 interactions. However, these effects were superseded by the significant three-way interaction among S1_type, group and age, *F*(2,897.27) = 3.04, *p* = .048. Bonferroni adjusted simple effects revealed that while at 2 months both groups were slower in disengaging visual attention from the non-rhythmic S1_type compared to the static and rhythmic S1_types (all *ps* < .001), at birth only the Experimental group displayed a similar trend, with the saccadic latencies in the non-rhythmic conditions being longer compared to both the static, t(896) = 3.67, p < .001  and rhythmic S1_types, t(913) = 3.91, p < .001. Between-group comparisons for each condition were not significant at either age (all *ps* > .18). No other comparisons reached significance (all *ps* >.17).

*lmer(log(SaccadicLatency)~ S1_Type*Age*PrenRhyEnr_index+(1|ID), data=DB_Exp)*

The model showed that the prenatal rhythmic enrichment index was involved in a significant duple interaction with age *F*(1,183.14) = 21.84, *p* < .001and in a triple interaction with age and S1_type, *F*(2, 378.81) = 4.28, *p* = .01. Bonferroni corrected simple trends confirmed that while at birth the effect of the amount of enrichment was negative in all the conditions (bs < -.007, *ps* < .037), at 2 months the amount of enrichment significantly impacted latencies only in the non-rhythmic S1_type, with a positive trend, b = .006, *t*(214) = 3.03, *p* = .003.

*lmer(log(SaccadicLatencies) ~ S1_type*Age* PrenRelax_index+(1|ID), data= DB_Con)*

This model yielded no effects involving the prenatal relaxation index (all *ps* > .2).

*lmer(log(SaccadicLatency) ~ S1_Type*PostRhyExp_index*Group + (1 | ID), data = DB_2m)*

Results showed no effects involving the postnatal rhythmic exposure index (all *ps* > .3).

**Statistical analyses with exact age as covariate**

*lmer(SaccadicLatency ~ S1_Type*Group + Age_hours+(1 | ID), data = DB_SacLat_Neo_age)*

The model showed no effect of Age_hours F(1,47.31) = .32, *p* = .57.

*lmer(SaccadicLatency ~ S1_Type*Group + Age_days+(1 | ID), data = DB_SacLat_2mo_age)*

The model showed no effect of Age_days F(1,55.5) = .2, *p* = .66.

*lmer(N_ValidTrials ~ S1_Type*Group + Age_hours+(1 | ID), data = DB_ValTrial_Neo_age)*

The model showed no effect of Age_hours F(1,54.79)=.05, *p*=.83.

*lmer(N_ValidTrials ~ S1_Type*Group + Age_days+(1 | ID), data = DB_ValTrial_2mo_age)*

The model showed no effect of Age_days F(1,51) = 1.07, *p* = .31.

**Statistical analysis with number of valid trials as covariate**

*lmer(mean_latency ~ S1_Type *Age*Group + N_ValidTrials +(1|ID), data = DB)*

The model showed no effect of *N_ValidTrials,* F(1,325.95) = .23, *p* = .63, while the critical *S1 Type × Group × Age* interaction remained significant, F(2, 233.46) = 6.95, *p* = .001.

**Results from Bayesian analyses**

**Number of valid trials**

*model_bayes_ValidTRials <- brm( N_ValidTrials ~ S1_Type * Group * Age + (1 | ID), data = DB_ValidTrials, family = poisson(), prior = my_priors, sample_prior = "yes", chains = 4, cores = 4, iter = 40000)*

*bf_fixed_ValidTrials <- bayesfactor_parameters(model_bayes_ValidTRials)*

| **Parameters** | **BF** |
| --- | --- |
| (intercept) | 1.36e+10 |
| S1_TypeRhythmic | .159 |
| S1_TypeStatic | .162 |
| GroupExperimental | .158 |
| AgeBirth | .150 |
| S1_TypeRhythmic:GroupExperimental | .237 |
| S1_TypeStatic:GroupExperimental | .204 |
| S1_TypeRhythmic:AgeBirth | .230 |
| S1_TypeStatic:AgeBirth | .240 |
| GroupExperimental:AgeBirth | .197 |
| S1_TypeRhythmic:GroupExperimental:AgeBirth | .282 |
| S1_TypeStatic:GroupExperimental:AgeBirth | .457 |

**Saccadic latencies**

*model_bayes_SacLat <- brm( SaccadicLatency ~ S1_Type * Group * Age + (1 | ID), data = DB_SacLat,*

*family = lognormal(), prior = my_priors, sample_prior = "yes", chains = 4, cores = 4, iter = 40000)*

*bf_fixed_SacLat <- bayesfactor_parameters(model_bayes_SacLat)*

| **Parameters** | **BF** |
| --- | --- |
| (intercept) | 1.61e+146 |
| S1_TypeRhythmic | 114.42 |
| S1_TypeStatic | 3.08e+04 |
| GroupExperimental | 0.116 |
| AgeBirth | 9.59e+03 |
| S1_TypeRhythmic:GroupExperimental | 0.207 |
| S1_TypeStatic:GroupExperimental | 0.127 |
| S1_TypeRhythmic:AgeBirth | 45.96 |
| S1_TypeStatic:AgeBirth | 29.59 |
| GroupExperimental:AgeBirth | 1.49 |
| S1_TypeRhythmic:GroupExperimental:AgeBirth | 1.33 |
| S1_TypeStatic:GroupExperimental:AgeBirth | 0.642 |

*model_prior <- unupdate(model_bayes)
contr_post <- emmeans(model_bayes, pairwise ~ S1_Type | Group * Age)
contr_prior <- emmeans(model_prior, pairwise ~ S1_Type | Group * Age)
bf_results <- bayesfactor_parameters(contr_post, prior = contr_prior)*

| **Parameters** | **BF** |
| --- | --- |
| Non-Rhythmic Control 2months | 8.81e+136 |
| Rhythmic Control 2months | 2.28e+111 |
| Static Control 2months | 2.76e+131 |
| Non-Rhythmic Experimental 2months | 2.07e+100 |
| Rhythmic Experimental 2months | 1.93e+85 |
| Static Experimental 2months | 3.33e+92 |
| Non-Rhythmic Control Birth | 1.86e+135 |
| Rhythmic Control Birth | 3.97e+144 |
| Static Control Birth | 5.83e+109 |
| Non-Rhythmic Experimental Birth | 3.84e+119 |
| Rhythmic Experimental Birth | 7.50e+117 |
| Static Experimental Birth | 3.53e+121 |
| (Non-Rhythmic) - Rhythmic Control 2months | 76.59 |
| (Non-Rhythmic) - Static Control 2months | 1.04e+04 |
| Rhythmic - Static Control 2months | Inf |
| (Non-Rhythmic) - Rhythmic Experimental 2months | 1.12e+03 |
| (Non-Rhythmic) - Static Experimental 2months | 7.27e+03 |
| Rhythmic - Static Experimental 2months | 0.021 |
| (Non-Rhythmic) - Rhythmic Control Birth | 0.083 |
| (Non-Rhythmic) - Static Control Birth | 0.070 |
| Rhythmic - Static Control Birth | 0.139 |
| (Non-Rhythmic) - Rhythmic Experimental Birth | 18.58 |
| (Non-Rhythmic) - Static Experimental Birth | 17.55 |
| Rhythmic - Static Experimental Birth | 0.014 |

*contr_post2 <- emmeans(model_bayes_SacLat, pairwise ~ S1_Type * Group | Age)
contr_prior2 <- emmeans(model_prior, pairwise ~ S1_Type * Group | Age)
bf_results2 <- bayesfactor_parameters(contr_post2, prior = contr_prior2)*

| **Parameters** | **BF** |
| --- | --- |
| Non-Rhythmic Control - Non-Rhythmic Experimental 2months | 0.112 |
| Rhythmic Control - Rhythmic Experimental 2months | 0.158 |
| Static Control – Static Experimental 2months | 0.056 |
| Non-Rhythmic Control - Non-Rhythmic Experimental Birth | 0.907 |
| Rhythmic Control - Rhythmic Experimental Birth | 0.135 |
| Static Control - Static Experimental Birth | 0.030 |

**Prenatal intervention metrics – Prenatal rhythmic enrichment index**

*model_bayes_PrenRhyEnrIndex <- brm(SaccadicLatency ~ S1_Type * PrenRhyEnr_index * Age + (1|ID),*

*data = DB_Exp, family = lognormal(), prior = my_priors, sample_prior = "yes", chains = 4, cores = 4, iter = 40000)*

*bf_fixed_PrenRhyEnrIndex <- bayesfactor_parameters(model_bayes_PrenRhyEnrIndex)*

| **Parameters** | **BF** |
| --- | --- |
| (intercept) | 2.62e+36 |
| S1_TypeRhythmic | Inf |
| S1_TypeStatic | 0.289 |
| PrenRhyEnr_index | 0.066 |
| AgeBirth | 2.00e+06 |
| S1_TypeRhythmic:PrenRhyEnr_index | 0.007 |
| S1_TypeStatic:PrenRhyEnr_index | 0.028 |
| S1_TypeRhythmic:AgeBirth | 0.827 |
| S1_TypeStatic:AgeBirth | 1.38 |
| PrenRhyEnr_index:AgeBirth | 25.69 |
| S1_TypeRhythmic:PrenRhyEnr_index:AgeBirth | 0.010 |
| S1_TypeStatic:PrenRhyEnr_index:AgeBirth | 0.052 |

*tr_post <- emtrends(model_bayes_PrenRhyEnrIndex, pairwise ~ Age, var = "PrenRhyEnr_index")
model_prior <- unupdate(model_bayes_PrenRhyEnrIndex)
tr_prior <- emtrends(model_prior, pairwise ~ Age, var = "PrenRhyEnr_index")
bf_results_PosthocPrenRhyEnr <- bayesfactor_parameters(tr_post, prior = tr_prior)*

| **Parameters** | **BF** |
| --- | --- |
| 2months | 0.004 |
| Birth | 0.190 |
| 2months - Birth | 6.01 |

**Prenatal intervention metrics – Prenatal Relaxation index**

*model_bayes_PrenRelaxIndex <- brm( SaccadicLatency ~ S1_Type * PrenRelax_index * Age + (1 | ID),*

*data = DB_Con, family = lognormal(), prior = my_priors, sample_prior = "yes", chains = 4, cores = 4, iter = 40000)*

*bf_fixed_PrenRelaxIndex <- bayesfactor_parameters(model_bayes_PrenRelaxIndex)*

| **Parameters** | **BF** |
| --- | --- |
| (intercept) | 6.92e+48 |
| S1_TypeRhythmic | 4.09 |
| S1_TypeStatic | 0.474 |
| PrenRelax_index | 0.001 |
| AgeBirth | 1.44 |
| S1_TypeRhythmic:PrenRelax_index | 0.001 |
| S1_TypeStatic:PrenRelax_index | 0.005 |
| S1_TypeRhythmic:AgeBirth | 0.359 |
| S1_TypeStatic:AgeBirth | 0.336 |
| PrenRelax_index:AgeBirth | 0.002 |
| S1_TypeRhythmic:PrenRelax_index:AgeBirth | 1.00e-252 |
| S1_TypeStatic:PrenRelax_index:AgeBirth | 0.008 |

**Postnatal exposure metrics**

*model_bayes_PostRhyExpIndex <- brm(SaccadicLatency ~ S1_Type * PostRhyExp_index * Group + (1|ID), data = DB_SacLat_2m, family = lognormal(), prior = my_priors, sample_prior = "yes", chains = 4, cores = 4, iter = 40000)*

*bf_fixed_PostRhyExpIndex <- bayesfactor_parameters(model_bayes_PostRhyExpIndex)*

| **Parameters** | **BF** |
| --- | --- |
| (intercept) | 2.46e+86 |
| S1_TypeRhythmic | 50.51 |
| S1_TypeStatic | 149.03 |
| PostRhyExp_index | 0.485 |
| GroupExperimental | 0.149 |
| S1_TypeRhythmic:PostRhyExp_index | 0.448 |
| S1_TypeStatic:PostRhyExp_index | 1.68 |
| S1_TypeRhythmic:GroupExperimental | 0.175 |
| S1_TypeStatic:GroupExperimental | 0.146 |
| PostRhyExp_index:GroupExperimental | 0.767 |
| S1_TypeRhythmic:PostRhyExp_index:GroupExperimental | 0.588 |
| S1_TypeStatic:PostRhyExp_index:GroupExperimental | 0.696 |

*tr_post <- emtrends( model_bayes_PostRhyExpIndex, pairwise ~ S1_Type, var = "PostRhyExp_index")*

*model_prior <- unupdate(model_bayes_PostRhyExpIndex)*

*tr_prior <- emtrends(model_prior, pairwise ~ S1_Type, var = "PostRhyExp_index")*

*bf_results <- bayesfactor_parameters(tr_post, prior = tr_prior);*

| **Parameters** | **BF** |
| --- | --- |
| *Non-Rhythmic* | *0.298* |
| *Rhythmic* | *0.197* |
| *Static* | *0.242* |
| *(Non-Rhythmic) - Rhythmic* | *0.313* |
| *(Non-Rhythmic) – Static* | *0.496* |
| *Rhythmic - Static* | *0.409* |
